# Crosstalk between auxin and gibberellin during stalk elongation in flowering Chinese cabbage

**DOI:** 10.1038/s41598-021-83519-z

**Published:** 2021-02-17

**Authors:** Erfeng Kou, Xinmin Huang, Yunna Zhu, Wei Su, Houcheng Liu, Guangwen Sun, Riyuan Chen, Yanwei Hao, Shiwei Song

**Affiliations:** College of Horticulture, South China Agricultural University, Guangzhou, China

**Keywords:** Auxin, Gibberellins, Plant hormones

## Abstract

Plant growth and development are tightly regulated by phytohormones. However, little is known about the interaction between auxin and gibberellin acid (GA) during flower stalk elongation and how it is directly related to organ formation. Therefore, the effects of indole acetic acid (IAA) and GA_3_ treatments and their interaction on flower stalk elongation in flowering Chinese cabbage were investigated. The growth of flowering Chinese cabbage is regulated by IAA and GA_3,_ and the opposite results were observed after treatments with uniconazole (GA synthesis inhibitor) and N-1-naphthylphthalamic acid (NPA) (auxin transport inhibitor). Anatomical analysis of the pith region in stalks revealed that IAA promoted expansion via signal transduction and transport pathways. GA_3_ regulated the elongation of flower stalks by controlling GA synthesis and partially controlling the IAA signaling pathway. GA_3_ also had a stronger effect on stalk elongation than IAA. The results of qRT-PCR and histological analysis revealed that GA_3_ and IAA induced the expansion of cell walls by activating the expression of genes encoding cell wall structural proteins such as Expansin (EXP). These findings provide new insights into the mechanism of stalk formation regulated by the combination of IAA and GA_3_.

## Introduction

*Brassica* spp. vegetables belong to the family Brassicaceae, which is one of the most successful plant families. There are a large number of *Brassica* vegetables cultivated and consumed worldwide^1,2^. Among these, flowering Chinese cabbage (*B*. *campestris*), Chinese kale (*B*. *alboglabra*), and purple cai-tai (*B*. *campestris*) are usually named cruciferous stalk vegetables because their edible parts are their elongated and expanded flower stalks (also called flower stems). These flower stalks are part of the major reproductive organs, which determine the yield and nutritional quality^3^. These are quite different from other Brassicaceae plants, e.g., cabbage and pakchoi. Therefore, it is particularly important to study organ formation and the regulatory mechanism of flower stalk formation in these vegetables. Until now, there has been little information regarding this topic.

Gibberellins (GAs), auxin (IAA), cytokinin (CTK), and brassinosteroid (BR) regulate stalk development through complex signal transduction pathways^4^. Bolting and flowering are significant reproductive processes and have been extensively researched in plants such as *Arabidopsis thaliana* and radish (*Raphanus sativus* L.)^5,6^. For example, IAA can induce internode elongation by regulating the production of active GA in pea^7,8^, and GA can regulate internode elongation by synthesis and transport of IAA in Arabidopsis^9^. IAA and GAs can promote cell division (proliferation), cell growth (expansion, elongation), differentiation, and ultimately control plant growth and development^10–12^. Both IAA and GA can induce cell elongation by activating cell wall structural proteins and enzymes, such as expansin (EXP), xyloglucan endotransglycosidase/hydrolase (XTH), and pectinesterase (PME), to alter cell wall polymer interactions^13–15^. GA synthesis and signaling pathways can affect internode elongation by regulating cell elongation and cell differentiation^16^. The interaction between auxin and GA was pointed out when the GA was discovered as a secondary plant growth hormone. In subsequent studies, the crosstalk of auxin and GA has been widely reported in different plant species. For instance, Ross et al. found that GA biosynthesis was enhanced by auxin in the stems of decapitated pea (*Pisum sativum* L.) and tobacco (*Nicotiana tabacum* L.). They also pointed out that the auxin was a link between the apical bud and biosynthesis of bioactive GA during internode expansion. It can be concluded that the crosstalk between auxin and GA in different crops varied. Even in the same plants, distinct organs showed the same contradictory results^17^.

Flowering Chinese cabbage (*Brassica campestris* L. ssp. *chinensis* var. *utilis* Tsen et Lee) is popular in south China and is one of the most important stalk vegetables. It is cultivated throughout China nowadays, owing to its high nutritional value, soluble fiber, and high vitamin C in the edible stalks^3^. Like *Arabidopsis thaliana* and radish, the bolting process of flowering Chinese cabbage includes stalk elongation and expansion. Our study also found that uniconazole treatment can inhibit the bolting of flowering Chinese cabbage without affecting flowering, suggesting that it has different bolting mechanisms than other cruciferous plants. However, previous studies have found IAA and GA peaks during stalk formation^18^. Low temperature could also significantly increase the GA, IAA, and CTK contents in stalk tips, thereby accelerating bolting in flowering Chinese cabbage^19^. Thus, IAA and GA might be involved in regulating the development of stalk vegetables. In addition, phytohormone signal transduction, cell expansion, and cell division are involved in stalk developmental processes^18^; however, there is limited systematic research about the mechanism of IAA and GA actions in stalk development. Further research is needed to clarify how IAA and GA link with morphological characteristics to regulate bolting in flowering Chinese cabbage. Therefore, the regulation mechanism between the formation of the swelled stalk and IAA supplemented with GA were carefully investigated in the present study.

## Results

### IAA-induced flower stalk elongation

**Figure 1:**
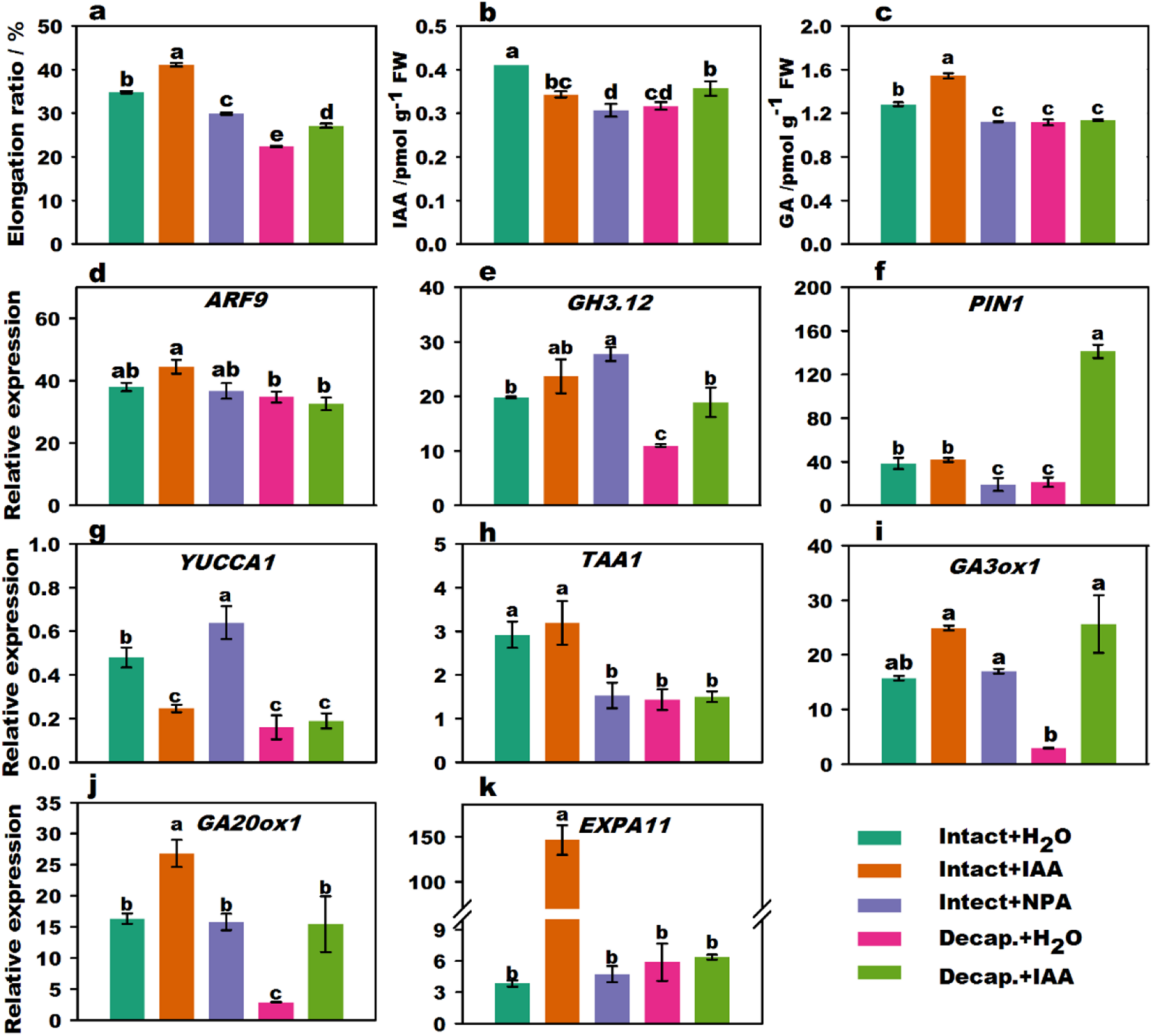
Effect of IAA treatments on stalk elongation, endogenous hormone content, and related gene expression of flowering Chinese cabbage. Stalk elongation ratio of flowering Chinese cabbage (**a**). Changes in endogenous IAA (**b**) and GA (**c**) content in stalk tips treated with different phytohormones. The relative expression of signal transduction (**d**,**e**) genes, polar transport genes (**f**), IAA biosynthesis (**g,h**), GA biosynthesis (**i**,**j**), and cell expansion (**k**)-related genes under different phytohormone treatments. Values with different letters indicate significant differences at *p* < 0.05, according to Duncan’s multiple range tests. Error bars indicate standard errors of three biological replicates. *Decap*. decapitation treatment.

Compared to intact plants, the stalk elongation of flowering Chinese cabbage was significantly inhibited by decapitation and NPA (N-1-naphthylphthalamic acid) treatments, whereas stalk elongation was stimulated by IAA treatment (Fig. 1a). The endogenous GA content in stalk tips of the intact plant increased with exogenous IAA treatment (Fig. 1b,c). After decapitation + IAA treatment, the IAA content was significantly higher than that after decapitation + H_2_O treatment. Interestingly, the IAA content in the intact plants + IAA treatment group was significantly lower than that of intact plants, which might be due to reduced endogenous IAA synthesis following application of exogenous IAA in plants. In addition, endogenous IAA and GA contents were significantly reduced in the intact plants + NPA treatment group compared to those in intact plants.

The expression of *ARF9* (*auxin response factor 9*), *GH3.12* (*4-substituted benzoates-glutamate ligase*), *PIN1* (*auxin efflux carrier component 1*), *YUCCA1* (*indole-3-pyruvate monooxygenase*), *TAA1* (*tryptophan aminotransferase 1*), *GA*3*ox1 (gibberellin 3-oxidase 1*), *GA20ox1* (*gibberellin 20 oxidase 1*), and *EXPA11* (*expansin-A11*) are shown in Fig. 1d–k. *YUCCA1*, *TAA1*, *GA20ox1*, and *GH3.12* expression was suppressed by decapitation compared to that in intact plants. However, the expression of *GA20ox1* and *EXPA11* (*expansin-A11*) was upregulated in the intact plants + IAA treatment group. Moreover, the expression of *GA20ox1*, *GA3ox1, GH3.12*, and *PIN1* (*auxin efflux carrier component 1*) was significantly upregulated with decapitation + IAA treatment compared to that with decapitation + H_2_O treatment. The expression of *PIN1* and *TAA1* in the NPA treatment group was also repressed. Thus, IAA stimulated the relative expression levels of auxin signal transduction-related genes and GA biosynthesis genes and induced a marked increase in GA content and stalk elongation.

### GA_3_ promotes flower stalk elongation

**Figure 2:**
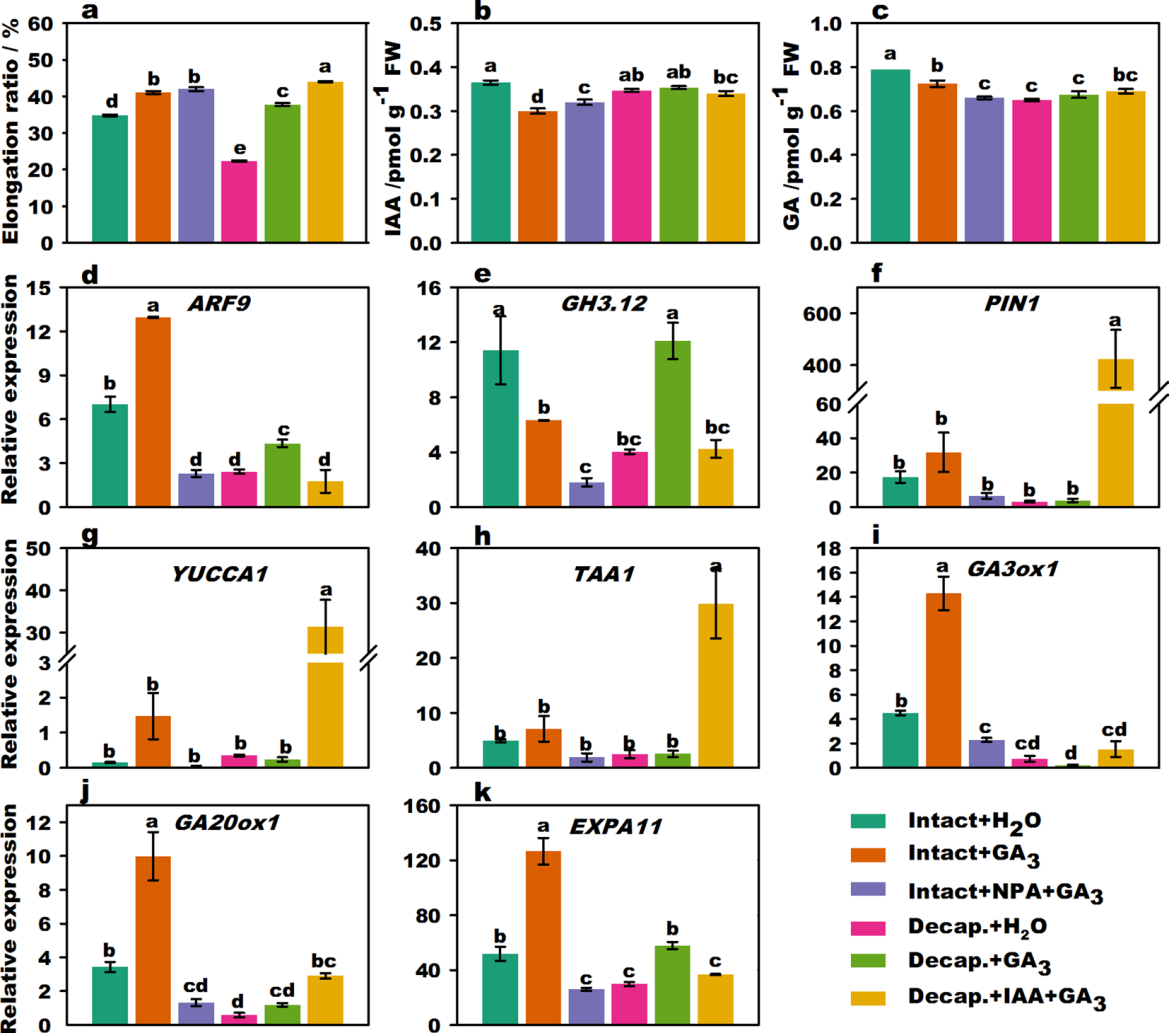
Effect of IAA and GA_3_ treatment on stalk elongation, endogenous hormone content, and related gene expression of flowering Chinese cabbage. Stalk elongation ratio of flowering Chinese cabbage (**a**). Changes in endogenous IAA (**b**) and GA (**c**) contents in stalk tips treated with different phytohormones. The relative expression of signal transduction (**d**,**e**) genes, polar transport genes (**f**), IAA biosynthesis (**g**,**h**), GA biosynthesis (**i**,**j**), and cell expansion (**k**)-related genes under different phytohormone treatments. Values with different letters indicate significant differences at *p* < 0.05, according to Duncan’s multiple range tests. Error bars indicate standard errors of three biological replicates.

Stalk elongation of flowering Chinese cabbage was significantly promoted (*p* < 0.05, Duncan’s multiple range tests) by exogenous GA_3_ treatment in both intact plants and decapitated plants (Fig. 2a). There were no significant differences between the intact + GA_3_ treatment group and the intact + NPA + GA_3_ treatment group, indicating that the decrease in elongation ratios following NPA treatment can be recovered by treatment with GA_3_. It was clear that the elongation ratio was the highest following decapitation + IAA + GA_3_ treatment. In addition, the elongation ratio following decapitation + IAA + GA_3_ (44.0%) treatment was significantly higher than that with both decapitation + GA_3_ (37.8%) and decapitation + IAA treatments (Fig. 1a) (27.2%), but lesser than the sum (65.0%) following decapitation + GA_3_ and decapitation + IAA treatments (Supplementary Fig. S6). This indicates that the promoting effects of IAA and GA_3_ interacted to affect stalk elongation. The endogenous IAA and GA contents of intact plant + GA_3_ in the stalk tips were significantly lower than those of intact plants + H_2_O treatment (Fig. 2b,c). The GA contents of intact plants + NPA + GA_3_ treatment were significantly lower than those of the intact plants + GA_3_ treatment, suggesting that NPA could affect GA biosynthesis.

The expression of *ARF9*, *GA*3*ox1*, *GA20ox1,* and *EXPA11* (Fig. 2d,f,i,j) was activated following treatment of intact plants with GA_3_ compared to that in intact plants subjected to H_2_O treatment. Furthermore, compared with the expression of genes in intact plants subjected to GA_3_ treatment, the expression levels of *ARF9*, *GH3.12* (Fig. 2e), *GA*3*ox1*, *GA20ox1*, and *EXPA11* (Fig. 2k) significantly decreased in the intact plants + NPA + GA_3_ treatment group. The expression of *YUCCA1* (Fig. 2g), *TAA1* (Fig. 2h), and *PIN1* was significantly upregulated compared to that with decapitation + GA_3_ treatment, whereas the expression of *ARF9* and *GH3.12* was downregulated with decapitation + IAA + GA_3_ treatment. These results show that the expression of genes related to IAA signaling, transport, and GA-synthesis could be partly promoted by GA_3_ and inhibited by both NPA treatment and decapitation treatments.

In summary, IAA and GA_3_ produced in the stalk tip were two crucial factors that promoted the stalk growth of flowering Chinese cabbage. Exogenous GA_3_ could upregulate the expression of key genes involved in GA biosynthesis and IAA signaling. GA_3_ promoted the biosynthesis of endogenous GA and induced stalk elongation. Thus, IAA and GA stimulated each other and worked together.

### IAA and GA integrated regulate flower stalk elongation

**Figure 3:**
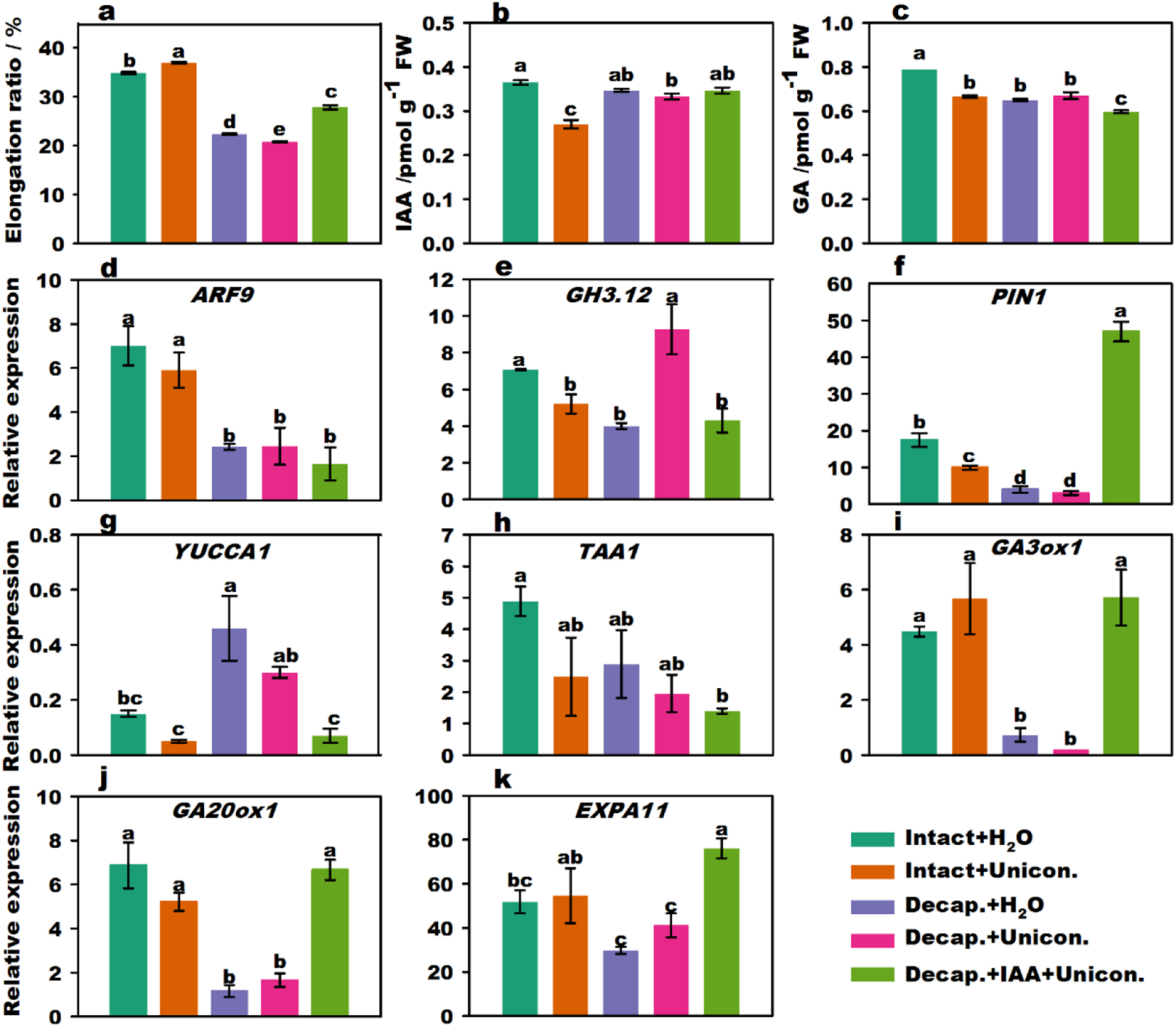
Effect of IAA and uniconazole treatment on stalk elongation, endogenous hormone content, and related gene expression of flowering Chinese cabbage. Stalk elongation ratio of flowering Chinese cabbage (**a**). Changes in endogenous IAA (**b**) and GA (**c**) content in stalk tips treated with different phytohormones. The relative expression of signal transduction genes (**d**,**e**), polar transport genes (**f**), IAA biosynthesis (**g**,**h**), GA biosynthesis (**i**,**j**), and cell expansion-related genes (**k**) under different phytohormone treatments. Values with different letters indicate significant differences at *p* < 0.05, according to Duncan’s multiple range tests. Error bars indicate standard errors of three biological replicates.

To further investigate the crosstalk between IAA and GA in the regulation of flower stalk elongation, uniconazole, a GA biosynthesis inhibitor was used. The elongation ratio in the decapitation + uniconazole treatment group was always lower than that in the decapitation + H_2_O treatment group (Fig. 3a and Supplementary Fig. S7), whereas the elongation ratio in the decapitation + IAA + uniconazole group was significantly higher than that in the decapitation + uniconazole treatment group. Thus, the elongation of stalks was inhibited by uniconazole and partially restored by IAA.

The endogenous GA and IAA contents in the intact plants + uniconazole treatment group were lower than those in intact plants alone (Fig. 3b,c). The IAA content with decapitation + uniconazole treatment was slightly lower than that with decapitation + H_2_O treatment; however, uniconazole did not affect endogenous GA levels. The endogenous IAA content with decapitation + IAA + uniconazole treatment was insignificantly higher than that with decapitation + uniconazole treatment, whereas the GA content declined considerably. The expression levels of *GH3.12*, *PIN1*, *YUCCA1*, *TAA1*, and *GA20ox1* (Fig. 3d–h,j) in the intact plants + uniconazole treatment group were lower than those in the intact plants + H_2_O treatment group. The expression levels of *PIN1*, *GA*3*ox1* (Fig. 3i), *GA20ox1*, and *EXPA11* (Fig. 3k) were significantly upregulated with decapitation + IAA + uniconazole treatment compared to those with decapitation + uniconazole treatment. Uniconazole + IAA treatment promoted partial GA synthesis and IAA-responsive gene expression corresponding with increased endogenous IAA content and partially recovered stalk growth. This means that there were interaction-promoted effects of IAA and GA on the elongation growth of flowering Chinese cabbage stalks.

### IAA and GA promote stalk elongation by regulating cell expansion

**Figure 4:**
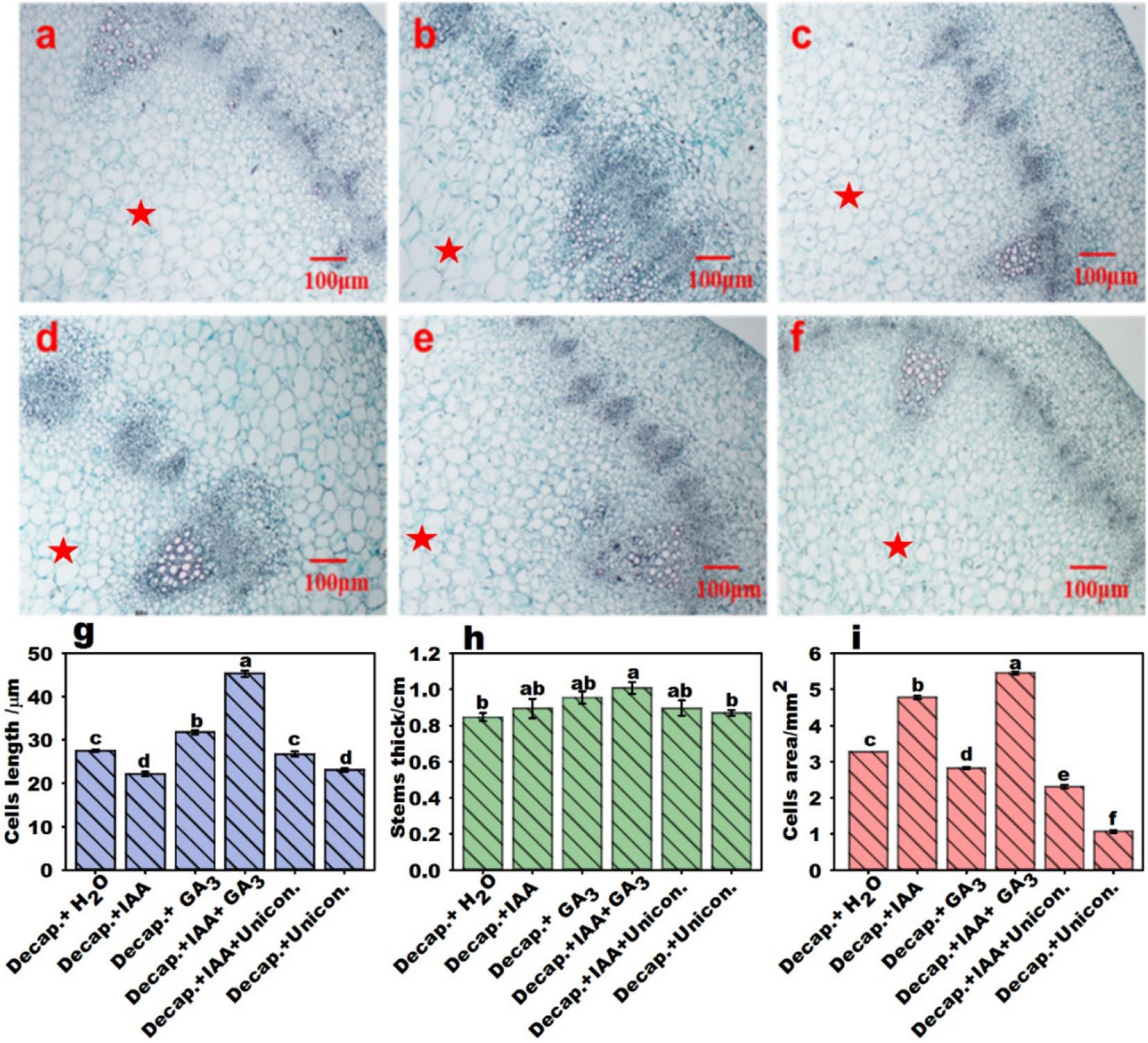
Stalk thickness and cell growth in flowering Chinese cabbage stalk tips following different treatments. Pictures show the pith cell microstructures of decapitated plant stalks in transverse sections: (**a**) decapitation + H_2_O, (**b**) decapitation + IAA, (**c**) decapitation + GA_3_, (**d**) decapitation + IAA + GA_3_, (**e**) decapitation + IAA + uniconazole, (**f**) decapitation + uniconazole. The stalk pith region was marked with a star (★). (**g**) Cell length from longitudinal sections of flowering Chinese cabbage stalks. (**h**) Diameter of stalk tips. (**i**) Cell area from transverse sections of flowering Chinese cabbage stalks. Values with different letters indicate significant differences at *p* < 0.05, according to Duncan’s multiple range tests. Error bars indicate standard errors.

The pith region occupies most of the flowering Chinese cabbage stalk, whose enlargement results from a rapid increase in pith tissue growth (Supplementary Fig. S1–S4). Measurements of stalk pith cells from transverse sections (Fig. 4a–f) and cell length from longitudinal sections were obtained after different treatments. The elongation of cells was promoted by GA_3_ treatment (Supplementary Fig. 4g), whereas IAA and uniconazole inhibited cell elongation. Cell length reached a maximum under the combined treatment with IAA and GA_3_. The stalk diameter was improved by IAA and GA_3_ treatments, and it reached a maximum with decapitation + GA_3_ + IAA treatment (Fig. 4h). The area of the apical stalk cells significantly increased with IAA treatment, whereas it decreased with GA_3_ treatment. The area of the cells in the decapitation + GA_3_ + IAA treatment group was maximized and significantly inhibited by uniconazole treatment (Fig. 4i).

## Discussion

### The role of IAA in flower stalk elongation

Thickening and elongation growth of stalks occur during stalk development in flowering Chinese cabbage. The apical meristem can produce different phytohormones, the distribution of which affects the occurrence and growth of organs; phytohormones thus play a crucial role in regulating plant growth^20^. Auxin can induce elongation in isolated stem segments and coleoptiles, and exogenous auxin treatment could promote stem elongation in auxin-deficient mutant plants^21,22^. Similarly, we found that decapitation treatment markedly inhibited stalk elongation, whereas IAA treatment promoted stalk elongation in decapitated plants (Fig. 1a). NPA, the polar transport inhibitor of IAA, inhibited the elongation of flower stalks. Therefore, our data showed that IAA, derived from the stalk apex, plays an important role in stalk growth and development in flowering Chinese cabbage.

*YUC1* and *TAA1* are two key genes in auxin biosynthesis^23,24^. The auxin signaling pathway is involved in hypocotyl elongation^25^. Auxin could promote the expression of auxin-responsive genes such as *Aux/IAA* and *GH3* transcription^26^. *ARF* is also involved in the growth of the inflorescence stem and flower organs. For instance, the growth of inflorescence stems was inhibited in *arf6* and *arf8* mutations^27^. The expression of *PIN1,* which plays a key role in auxin polar transport^28^, was down-regulated with NPA treatment^29,30^. In the present study, the expression of genes related to IAA signal transduction was upregulated with IAA treatment (Fig. 1). Thus, exogenous IAA may regulate the stalk elongation in flowering Chinese cabbage via regulating genes related to the transportation pathways of auxin.

### Crosstalk between IAA and GA in flower stalk elongation

One of the main biological functions of GA is to promote plant growth. Inhibition of endogenous GA synthesis leads to a dwarf phenotype^31^, whereas an increase of GA content in plants can promote plant stem elongation^32^. *GA20ox1* and *GA3ox1* were involved in the generation of bioactive GA. Ross et al. have reported that the biosynthesis of GA1 and expression of *GA3ox1* were reduced in decapitated stems^7^. The application of IAA can recover both transcript and GA1 biosynthesis^33^. In this study, both *GA20ox1* and *GA3ox1* expression and auxin and GA levels were repressed by decapitation. In addition, the application of IAA recovered the expression of GA20ox1 and *GA3ox1*, and consequently, the GA synthesis was also rehabilitated. Thus, it can be concluded that IAA, produced by the apical meristem, can induce GA production in the stalk of flowering Chinese cabbage, promote the *GA20ox1* expression levels, and stimulate internode elongation^7,32^. The inhibition of NPA in stalk elongation (Fig. 1a) was similar to maize^34^. The repressive effect of NPA was restored by a GA_3_ and NPA mixture (Fig. 2a), and GA_3_ can fully restore stalk elongation even with low IAA content. Therefore, GA_3_ might work downstream of IAA. Uniconazole can inhibit stalk elongation (Fig. 3a and Supplementary Fig. S7), while an IAA and uniconazole mixture cannot fully restore stalk elongation (Fig. 3a). The above results indicate that GA_3_ plays a more important role than IAA in stalk elongation. IAA might regulate the elongation of stalks in two pathways. First, IAA promotes stalk elongation with an increased biosynthesis of GA. Second, IAA may directly promote stem elongation with promoting some IAA signal transduction and transport genes. GA_3_ can also affect genes related to the IAA signaling and transport pathways; therefore, both GA_3_ and IAA are involved in the regulation of stalk development. The effect of crosstalk among plant hormones has been widely reported by scientists. BR and CTK even other phytohormone may also participate in the regulation of the growth of flowering Chinese cabbage stalks independently and in combination to GA and IAA. However, the specific biological functions of other phytohormones, such as BR, in stalk development need further investigation in future studies.

### Stimulated stalk elongation through mediated cell growth

Cell differentiation is involved in the bolting process of tuber mustard and cabbage^35,36^. In this study, IAA treatment increased the expression of some cell expansion genes leading to the promotion of cell expansion (Figs. 1k, 4i), but failed to promote cell elongation (Fig. 4g). Our previous study found that IAA promoted cell expansion and cell division in the flower stalks of flowering Chinese cabbage^18^. The auxin transcription factor (ARF) can affect stolon development by regulating cell division and expansion^11,37,38^. Therefore, IAA may promote stalk thickening and elongation by promoting cell expansion. GA can induce cell wall expansion by inducing the expression of expansion genes and xyloglucan endotransglycosylase/hydrolase genes^13,14^. The GA biosynthesis inhibitor, paclobutrazol, inhibits the expression of *EXPs*^39^. Exogenous GA_3_ treatment significantly upregulated the expression of the cell expansion-associated gene *EXPA11* and promoted cell elongation (Figs. 2f, 4g).

**Figure 5:**
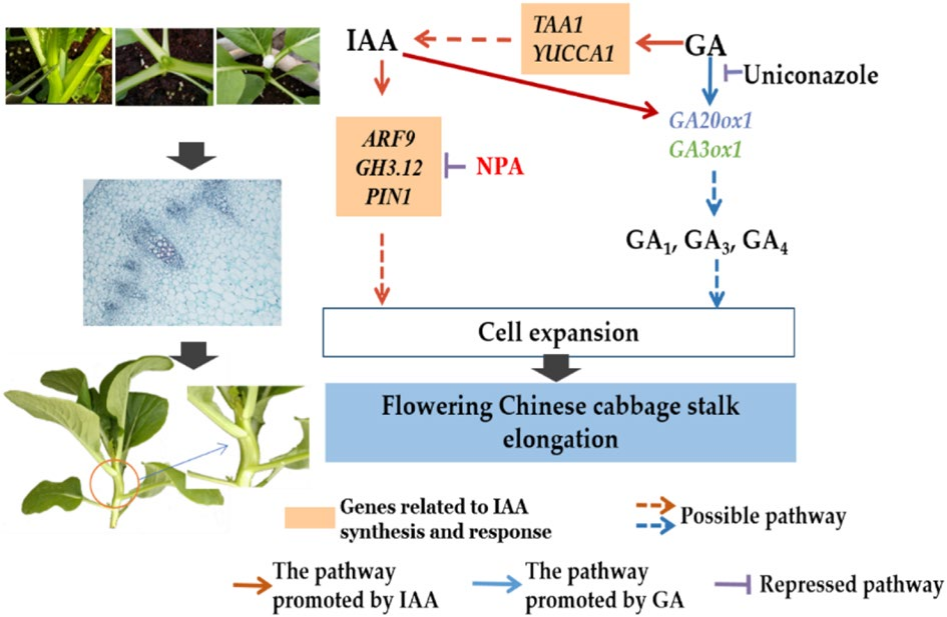
A hypothetical model of IAA and GA-stimulated stalk elongation in flowering Chinese cabbage.

In summary, a regulatory network can be hypothesized (Fig. 5). Exogenous IAA can regulate the expression of the auxin transport gene *PIN1* and signal transduction genes *ARF9* and *GH3.12*. NPA inhibition of *PIN1* expression can affect auxin transport. Exogenous GA_3_ can regulate GA production by regulating *GA20ox1* and *GA*3*ox1* expression, while uniconazole can inhibit GA biosynthesis. IAA and GA_3_ regulate stalk elongation together by regulating cell expansion.

## Conclusions

Exogenous IAA induced the elongation of stalks with the promoting of cell expansion and enhancing endogenous GA and IAA contents. Meanwhile, exogenous GA promoted stalk elongation partially with the increase of genes related to the IAA signal transduction and GA biosynthesis pathway. Anatomical findings showed that cell elongation and cell expansion were the main factors promoting stalk elongation in flowering Chinese cabbage. IAA and GA regulate the elongation of the stalk together, with GA being the main controlling factor for regulating the elongation of flowering Chinese cabbage stalks. Thus, our findings provide a basis for future studies on stalk development mechanisms in cruciferous stalk vegetables.

## Materials and methods

### Plant materials and treatments

Experiments were performed in a greenhouse located at South China Agricultural University (Guangzhou, China). The cultivar of flowering Chinese cabbage used was “Youqing 33 caixin” (Guangdong Suimei Agricultural Technology Co., Ltd, China). Seeds were sown in plug trays, using perlite as the substrate. Seedlings were grown until the third true leaf was fully expanded. Then, seedling plugs were transplanted into a non-woven culture bag (30 cm diameter × 20 cm height) filled with a mixed media consisting of peat, perlite, and coco peat (volume ratio of 3:1:2). All plants were watered every three days with an equal volume of Hoagland’s solution at a half-dose concentration.

In this study, 12 treatments were applied: Intact plant (intact) + H_2_O; decapitation (Decap.) + H_2_O; Intact + IAA; Decap. + IAA; Intact + NPA; Intact + GA_3_; Decap. + GA_3_; Intact + NPA + GA_3_; Decap. + IAA + GA_3_; Intact + uniconazole; Decap. + uniconazole; and Decap. + IAA + uniconazole. For this study, we divided these treatments into three groups. Each treatment had three replicates, which consisted of 10 plants. The three groups were as follows: (1) decapitation treatment: the tip of the stalk was cut with sharp-billed tweezers, and a piece of cotton ball containing different treatment solutions (H_2_O, IAA, GA_3_) was placed on the flat cut surface. (2) NPA treatment: the NPA was applied as a horizontal ring on the plant below the tip of the stalk. (3) Treatment of intact plants was carried out by spraying the solutions. All treatments were applied every 6 and 12 h in the day and at night, respectively, until it reached 60 h during the rapid bolting period (7**–**9 leaves).

The optimal IAA treatment concentration was 20 mg/L, according to our preliminary experiments (Supplementary Fig. S5). A GA_3_ concentration of 200 mg/L and 10 mg/L of uniconazole was used, as per our previous research^17^, and 2.91 mg/L of NPA was used, according to a previous study^40^.

### Elongation ratio and stalk diameter

Plant height at different treatment time (from the cotyledon to the shoot tip) was measured using a ruler to calculate stalk elongation. Elongation (%) = [(the length after treatment − the length before treatment)/the length before treatment] × 100. The images were examined under a digital microscope (VHX-5000; Keyence, Osaka, Japan), and the cell area and length were measured using Image-Pro plus 6 software (USA).

### RNA extraction, cDNA synthesis, and qRT-PCR

Shoot tips (5 mm) of flowering Chinese cabbage in different treatment groups were used for RNA extraction after treating for 1 h. Total RNA was extracted with Eastep™ Super Total RNA Extraction Kit (Promega Biotech Co., Ltd, Beijing, China) according to the manufacturer's instructions. RNA integrity was analyzed on a 1% agarose gel and in a 2100 Bioanalyzer (Agilent Technologies, Santa Clara, CA, USA). The extracted RNA samples were treated with DNase I (Promega Biotech Co., Ltd, Beijing, China), and cDNA was synthesized from 1 μg of total RNA using a reverse transcription mix (Promega Biotech Co., Ltd, Beijing, China). The qRT-PCR was performed as previously reported^17^, and the gene transcript levels were normalized to GAPDH expression. Gene-specific primers for qRT-PCR are listed in Supplementary Table S1.

### Paraffin sections and imaging

After 60 h of treatment, the stalk tips (5 mm) of flowering Chinese cabbage were used for sections and imaging samples. Cell length, cell area, and paraffin sections were analyzed as previously described^17^.

### Determination of IAA and GA content

The shoot tips (3 mm) of six plants were pooled into a single sample at 36 h after treatment, immediately flash-frozen in liquid nitrogen, and stored at − 80 °C. Three biological replicates were prepared for each treatment. The GA and IAA contents were determined by enzyme-linked immunosorbent assay (ELISA), and the ELISA kit was purchased from Shanghai Biotechnology Co., Ltd.

### Statistical analyses

Data were analyzed using SPSS 16 software (SPSS Inc. Chicago, IL, USA). The value with different letters was considered significantly different at *p* < 0.05. The experiments were repeated in triplicate, and the data shown are mean values with standard error (S.E.). Consistent elongation results have been obtained by repeated experiments carried out in different seasons. Although the differences shown in the figures were tiny, these treatments' effects are repeatable and reliable. Sigma Plot 11 software (SYSTAT, San Jose, CA, USA) was used to display data.

## Supplementary Information

Supplementary Information.
