# Supplementary material for: Crosstalk between auxin and gibberellin during stalk elongation in flowering Chinese cabbage
Source: Sci Rep. 2021 Feb 17;11:3976. doi: 10.1038/s41598-021-83519-z (PMC7889655; doi:10.1038/s41598-021-83519-z)
Supplement: Supplementary file 1 — Supplementary Information. [file 41598_2021_83519_MOESM1_ESM.pdf]

Crosstalk between auxin and gibberellin during stalk elongation in flowering Chinese cabbage

Erfeng Kou, Xinmin Huang, Yunna Zhu, Wei Su, Houcheng Liu, Guangwen Sun, Riyuan Chen, Yanwei Hao * and Shiwei Song*

College of Horticulture, South China Agricultural University, Guangzhou, China; kef@stu.scau.edu.cn (E.K.); hxmscau@163.com (X.H.); Zhuyn326@126.com (Y.Z.); susan_l@scau.edu.cn (W.S.); liuhch@scau.edu.cn (H.L.); sungw1968@scau.edu.cn (G.S.); rychen@scau.edu.cn (R.C.);

* Correspondence: yanweihao@scau.edu.cn (Y.H.); swsong@scau.edu.cn (S.S.).

Supporting Information

Table S1 Primer sequences

Primer name	Primer sequence（5′ －3′ ）
GAPDH	F:CAGGTTTGGAATTGTCGAGG
	R:GAGCTGTGGAAGCACCTTTC
EXPA11	F:GAACTTCGCTCTCCCCAACA
	R:TTCCGATCTTCTCCCAAGCG
TAA1	F:CACTCGGATCTTATCCTGCG
	R:CGTTCACCTGCTCTGCTCATA
GA3ox1	F:TCTAGCTGCCCATACCGACT
	R:GCGGTGTAGCACGCTCTTA
ARF9	F:GTATGAAGAGCTGTGGAAGCTA
	R:GGAGGAAGATCGAAAAGAGGCT
ARF8	F:ACAACAACCTGACCGTGTGT
	R:ACAGGTGGCTACTACTATCTCCA
PME3	F:CAGGGGCTGGAACTGCAAAT
	R:CGAGCGAAAAAGGAAAACCG
YUCCA1	F:TCACGAAGGAAGGGATGCCA
	R:CACTGGTCAGCTATTTCCCCG
EXPA8	F:TGTTCGTCCCGTAACCTTGG
	R:GGAACTAATGGGGACGACGG
GH3.12	F:CAGAGCTTAGTGCATAGCCGA
	R:CGAATGGAGAACCCTCGGAC
Aux/IAA1	F:ATGTCGGCGGGAGTGAGT
	R:GCTAGAGGGGTCGTACTCGT

PIN1	F:TCGATTTATCCACCGACCCA
	R:GTGTTGCTCATTCGTAGCGG
GA20ox1	F:CGCCGCCATTTCTGATGTTT
	R:GCATCCCCTCGCAATACTCA

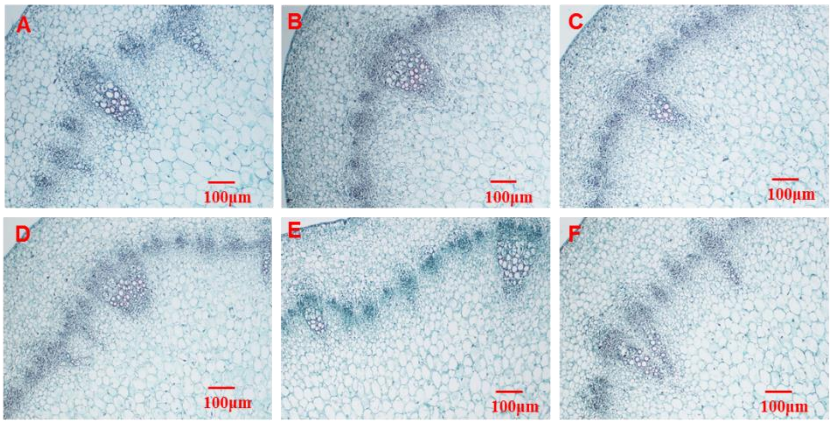

Figure S1. Pith cell microstructures of intact plant stalks in transverse sections. A: Intact + H_2O, B: Intact + IAA, C: Intact + NPA, D: Intact + NPA + GA_3, E: Intact + GA_3, F: Intact + Uniconazole.

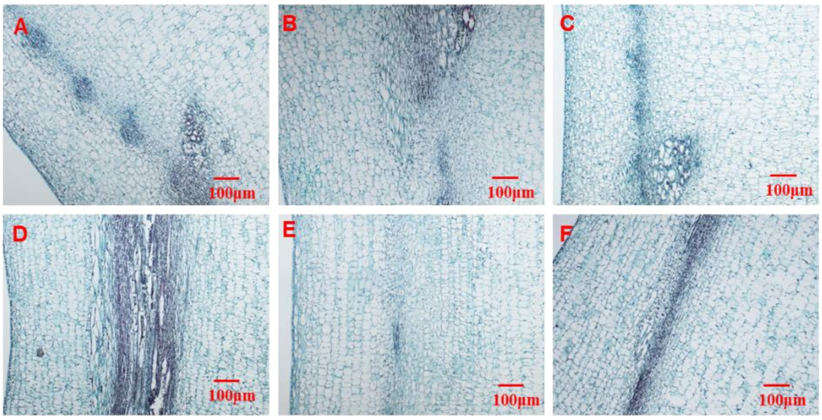

Figure S2. Pith cell microstructures of intact plant stalks in longitudinal sections. A: Intact + H_2O, B: Intact + IAA, C: Intact + NPA, D: Intact + NPA + GA_3, E: Intact + GA_3, F: Intact + Uniconazole.

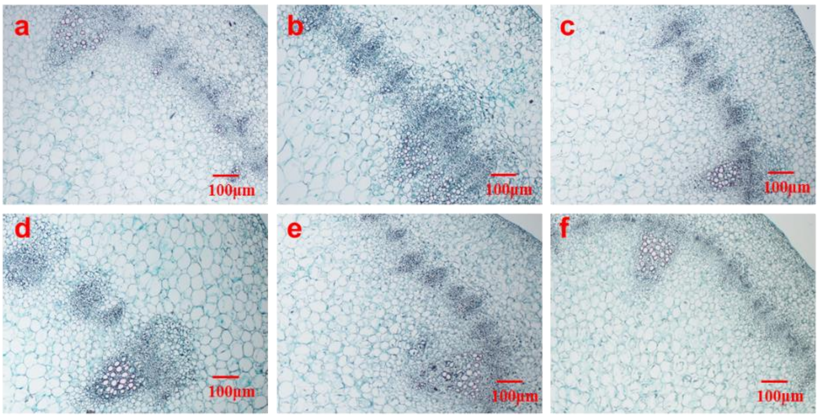

Figure S3. Pith cell microstructures of decapitated plant stalks in transverse sections. a: Decap. + H_2O, b: Decap. + IAA, c: Decap. + GA_3, d: Decap. + IAA + GA_3, e: Decap. + IAA + Unicon., f: Decap. + Unicon

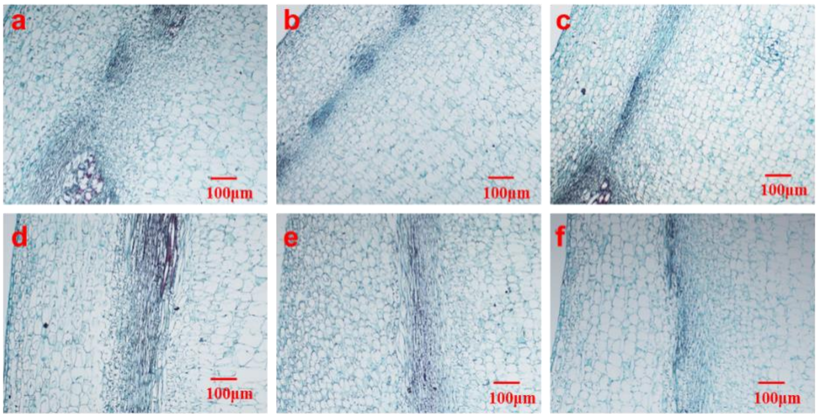

Figure S4. Pith cell microstructures of decapitated plant stalks in longitudinal sections. a: Decap. + H_2O, b: Decap. + IAA, c: Decap. + GA_3, d: Decap. + IAA + GA_3, e: Decap. + IAA + Unicon., f: Decap. + Unicon

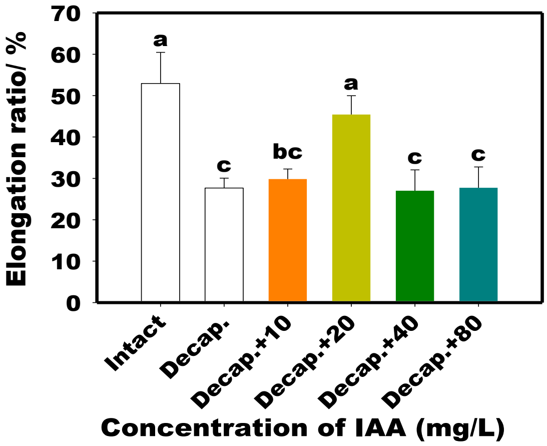

Figure S5. Stalk elongation ratios of flowering Chinese cabbage after treatment for 36 h with different IAA concentrations.

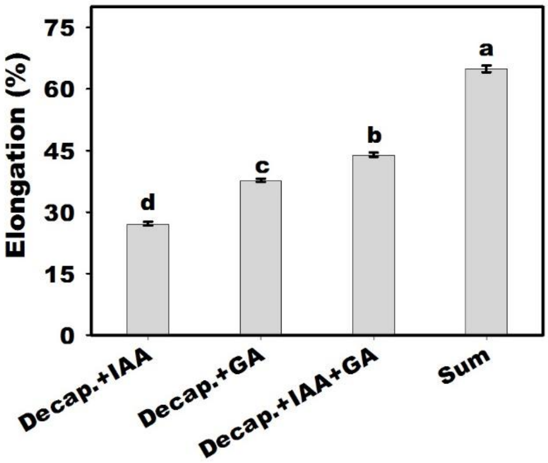

Figure S6. Stalk elongation ratios of flowering Chinese cabbage with different treatment in 60 h. Values with different letters indicate significant differences at $p < 0.01$ according to Duncan's multiple-range tests. Error bars indicate standard errors of three biological replicates.

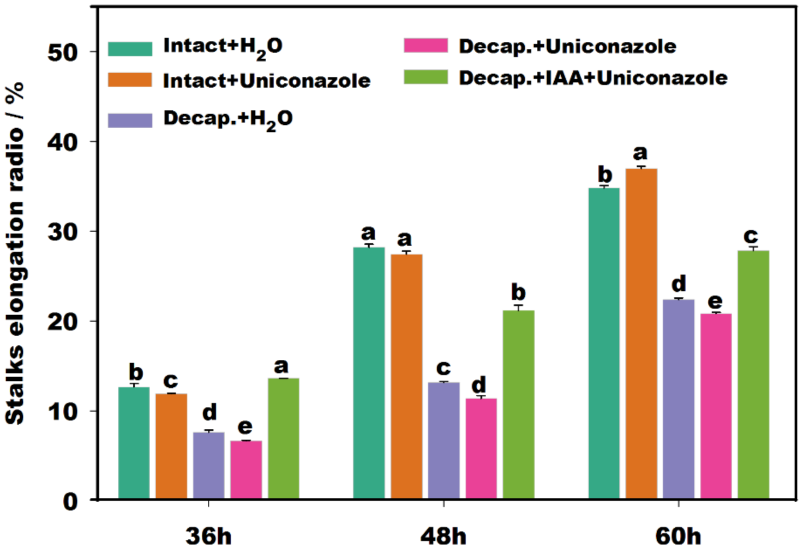

Figure S7. Stalk elongation ratios of flowering Chinese cabbage with different treatment in 36, 48, 60 h. Values with different letters indicate significant differences at $p < 0.01$ according to Duncan's multiple-range tests. Error bars indicate standard errors of three biological replicates.
